# Monitoring of anesthetic depth and EEG band power using phase lag entropy during propofol anesthesia

**DOI:** 10.1186/s12871-020-00964-5

**Published:** 2020-02-26

**Authors:** Hye Won Shin, Hyun Jung Kim, Yoo Kyung Jang, Hae Sun You, Hyub Huh, Yoon Ji Choi, Seung Uk Choi, Ji Su Hong

**Affiliations:** 1 Department of Anesthesiology and Pain Medicine, Korea University Anam Hospital, College of Medicine, Korea University, Goryodae-ro 73, Seongbuk-gu, 02841 Seoul, Republic of Korea; 2 Department of Anesthesiology and Pain Medicine, Ewha University Magok Hospital, College of Medicine, Ewha University, Seoul, Republic of Korea; 3 Department of Anesthesiology and Pain Medicine, Korea University Ansan Hospital, College of Medicine, Korea University, Gyeonggi-do, Republic of Korea

**Keywords:** Anesthetic depth monitoring, Bispectral index, Phase lag entropy, Propofol

## Abstract

**Background:**

Phase lag entropy (PLE) is a novel anesthetic depth indicator that uses four-channel electroencephalography (EEG) to measure the temporal pattern diversity in the phase relationship of frequency signals in the brain. The purpose of the study was to evaluate the anesthetic depth monitoring using PLE and to evaluate the correlation between PLE and bispectral index (BIS) values during propofol anesthesia.

**Methods:**

In thirty-five adult patients undergoing elective surgery, anesthesia was induced with propofol using target-controlled infusion (the Schneider model). We recorded the PLE value, raw EEG, BIS value, and hemodynamic data when the target effect-site concentration (Ce) of propofol reached 2, 3, 4, 5, and 6 μg/ml before intubation and 6, 5, 4, 3, 2 μg/ml after intubation and injection of muscle relaxant. We analyzed whether PLE and raw EEG data from the PLE monitor reflected the anesthetic depth as the Ce of propofol changed, and whether PLE values were comparable to BIS values.

**Results:**

PLE values were inversely correlated to changes in propofol Ce (propofol Ce from 0 to 6.0 μg/ml, r^2^ = − 0.83; propofol Ce from 6.0 to 2.0 μg/ml, r^2^ = − 0.46). In the spectral analysis of EEG acquired from the PLE monitor, the persistence spectrogram revealed a wide distribution of power at loss of consciousness (LOC) and recovery of consciousness (ROC), with a narrow distribution during unconsciousness. The power spectrogram showed the typical pattern seen in propofol anesthesia with slow alpha frequency band oscillation. The PLE value demonstrated a strong correlation with the BIS value during the change in propofol Ce from 0 to 6.0 μg/ml (r^2^ = 0.84). PLE and BIS values were similar at LOC (62.3 vs. 61.8) (*P* > 0.05), but PLE values were smaller than BIS values at ROC (64.4 vs 75.7) (*P* < 0.05).

**Conclusions:**

The PLE value is a useful anesthetic depth indicator, similar to the BIS value, during propofol anesthesia. Spectral analysis of EEG acquired from the PLE monitor demonstrated the typical patterns seen in propofol anesthesia.

**Trial registration:**

This clinical trial was retrospectively registered at ClinicalTrials.gov at October 2017 (NCT03299621).

## Background

Level of consciousness is related to the complexity and variability of communication between the brain regions [1]. The diverse functional connectivity of the brain in awake state is diminished during anesthesia [2]. There are increases in the phase synchronization or shifts between electroencephalography (EEG) signals of the frontal brain during anesthesia, indicating a reduction in communication diversity [3]. The state of consciousness is more closely related to the temporal dynamics of the functional network configuration than to the strength of static connectivity [4, 5]. The processed EEG signal is an integral part of the brain function monitors used to measure the level of consciousness during anesthesia [6].

Unconsciousness is a fundamental component of general anesthesia; however, anesthesiologists have no reliable way of confirming that a patient is unconscious. Generally, loss of consciousness (LOC) is marked by an increase in low-frequency (< 1 Hz) EEG power, the loss of spatially coherent occipital alpha (8–12 Hz) oscillations, and the appearance of spatially coherent frontal alpha oscillations [7]; these dynamics are then reversed during recovery of consciousness (ROC) [8]. There have been reports of changes in functional connectivity and disruptions of frontal EEG communication in the brain during anesthesia with propofol [4, 9, 10], sevoflurane [10–12], and ketamine [10].

The Bispectral Index™ (BIS™, Aspect Medical Systems, USA), the most widely used monitor in clinical practice, is based on spectral analysis of frequency powers from one-channel EEG [4, 13, 14]. BIS™ is useful for titration of anesthetics and postoperative recovery [15, 16]. However, BIS monitors cannot provide information regarding functional connectivity in the brain. Previous studies have reported a poor correlation between BIS and depth of anesthesia or sedation [6, 13, 17].

Phase lag entropy (PLE) is an EEG-based anesthetic depth indicator that calculates diversity in temporal patterns of the phase relationship in the brain [4, 10]. The recently developed PLE monitor (PLEM™, Inbody Co., Ltd., Republic of Korea), which measures the PLE value, is a four-channel EEG anesthetic depth monitoring device [4, 18–20].

The purpose of this study was to evaluate the clinical performance of the PLEM™ to monitor anesthetic depth and to evaluate the correlation between PLE and BIS values during propofol anesthesia.

## Methods

The study was approved by an institutional review board (Korea University Anam Hospital, Institutional Review Board) (IRB No. 2017AN0268), and was prospectively registered, prior to patient enrollment, at ClinicalTrials.gov (NCT03299621, date of registration: October 2017). We also obtained written informed consent from all patients participating in the trial. This study used a prospective, observational, one-group design. The primary end-point of this study was to evaluate the clinical performance of the PLEM™ to monitor anesthetic depth during propofol anesthesia. The secondary end-point was to evaluate the correlation between the PLE and BIS values during propofol anesthesia.

### Anesthesia and monitoring

Thirty-five adult patients undergoing elective surgery under general anesthesia were enrolled for the study. Patients were aged 20–60 years with an American Society of Anesthesiologists (ASA) physical status I or II. Exclusion criteria were presence of cardiovascular disorders, cerebrovascular disorders, respiratory disorder, and an anticipated difficult airway. Table 1 summarizes the patient characteristics. All patients were premedicated with glycopyrrolate 0.2 mg intramuscularly 1 h before induction of anesthesia. In the operating room, routine standard monitoring was followed, including electrocardiogram, non-invasive blood pressure monitoring, pulse oximetry, capnography, and temperature monitoring. General anesthesia was induced using a propofol target-controlled infusion (TCI, Orchestra®, Fresenius Kabi, France), and intravenous (iv) rocuronium 0.9 mg/kg was administered for tracheal intubation. To maintain propofol TCI, we used a “staircase” TCI that automatically reached the target effect-site concentrations (Ce) after the propofol Ce was set. After endotracheal intubation, we maintained controlled ventilation with a tidal volume of 6–10 ml/kg, respiration rate of 10–12/min, and inspired oxygen concentration of 0.5. Anesthesia was maintained with remifentanil Ce 0–10 ng/ml (Minto model) and propofol Ce 0–6 μg/ml (Schneider model). Hemodynamics were maintained within a 20% range of baseline value using fluids, phenylephrine 100–200 μg iv (< 20% from baseline blood pressure), or hydralazine 5–10 mg iv (> 20% from baseline blood pressure).

**Table 1:** Characteristics of included patients

Patient characteristics (*n* = 35)	
Age (years)	38.3 ± 12.2
Male/Female (number)	21/14
Weight (kg)	69.9 ± 12.5
Height (cm)	168.7 ± 9.6
ASA I/ASA II (number)	23/12
Duration of anesthesia (min)	66.1 ± 47.5
Type of surgery	
Orthopedic surgery	24
Genitourinary surgery	5
Breast surgery	2
Gynecologic surgery	4

Data are mean ± standard deviation or number of patients. American Society of Anesthesiologists (ASA) physical status I; A normal healthy patient, ASA physical status II; A patient with mild systemic disease

### Preparation of the PLEM™ and BIS™ sensor

**Fig. 1:**
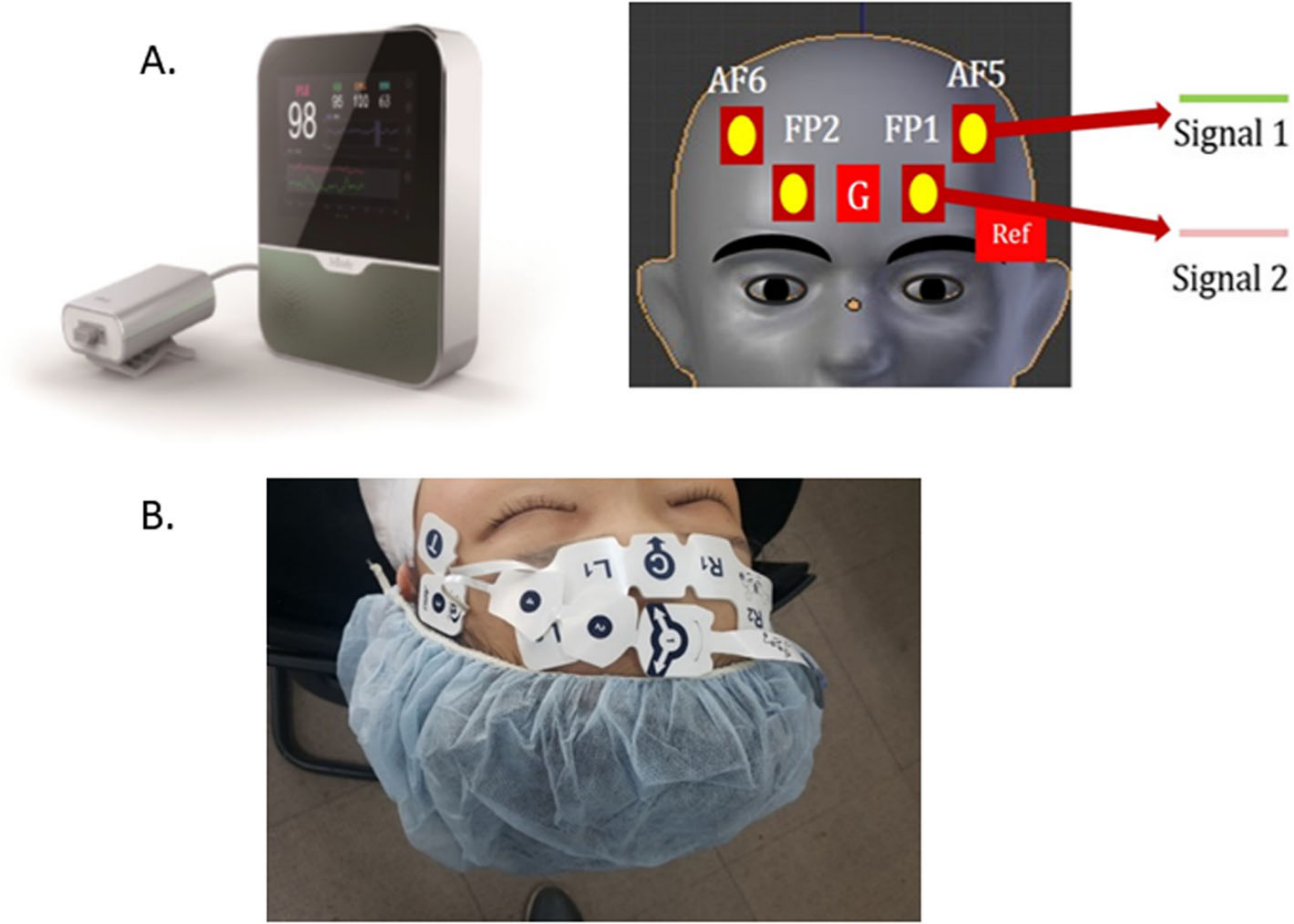
Illustration of electroencephalogram (EEG) channels and phase difference sign measurement. **a** PLEM™ measures the phase lag entropy (PLE) value. The PLE electrode array consists of electrodes positioned approximately at FP1 (L_1_), FP2 (R_1_), AF5 (L_2_), and AF6 (R_2_). The ground electrode is at F_PZ_ (G), and the reference electrode is at T3 of the temporal area of the face (T). **b** Both sensors are placed on the left temporal-frontal area with the bispectral index (BIS) sensor placed above the PLE sensor. Permitted for the copyright for PLEM™ monitor image [15, 01, 2020] by Copyright Holder (InBody co., ltd)

The PLEM™ and BIS™ were placed on the left temporal-frontal area, with the BIS™ sensor above the PLEM™ sensor as recommended by the manufacturer (Fig. 1). Both monitors displayed PLE and BIS values and trends, as well as electromyography (EMG) recordings of the forehead muscle, signal quality index (SQI), and real-time EEG waveforms. The anesthesiologist maintained an SQI > 70 for both devices to ensure accuracy of PLE and BIS values. Both PLEM™ and BIS™ monitors provide a calculated numeric PLE or BIS value between 0 (isoelectric EEG) and 100 (patient fully awake). The smoothing rates of the PLEM™ and was BIS™ were 4 s and 10 s, respectively.

### Data collection and EEG acquisition

Frontal raw EEG signals were recorded using the PLEM™ sensor. EEG data were recorded with a preamplifier bandwidth of 0.5–45 Hz and sampling rate of 128 Hz. In order to minimize noise in the EEG signal, we analyzed the data after filtering it through baseline correction and reducing non-specific artifacts. The PLEM™ electrode array was configured with electrodes positioned approximately at FP1 (L_1_), FP2 (R_1_), AF5 (L_2_), and AF6 (R_2_). The ground electrode was at Fpz, and the reference electrode was at position T3 on the temporal area of the face (T) (Fig. 1). Electrode impedance was less than 7 kΩ in each channel.

### Data measurement at time points

We collected data regarding patient demographics and anesthetic management. We acquired the data for PLE value, EEG band power, and BIS value using a USB memory card on PLEM™ and BIS™ devices at the following points in time: (1) prior to intubation as propofol Ce increased from 0 μg/ml to 2, 3, 4, 5, and 6 μg/ml, and after intubation as propofol Ce decreased from 6 μg/ml to 5, 4, 3, and 2 μg/ml; (2) two min after muscle relaxant injection and tracheal intubation; (3) at LOC, defined as when a patient no longer responds to the verbal command “open your eyes” (modified Observer’s Assessment Alertness/Sedation [OAA/S] scale = 2) repeated every 10 s during induction; and (4) at ROC, defined as when a patient once again obeys the verbal command “open your eyes” (modified OAA/S scale = 3) repeated every 10 s during emergence from anesthesia.

### Calculation of PLE

The PLE value was calculated following the approach used in Lee et al.’s study [4]. The degree of communication between different areas of the brain is correlated with the phase relationship among multi-channel EEG signals [8, 15]. In order to calculate the PLE value, the data from the frontal and prefrontal lobes were recorded using PLEM™ sensor electrodes arranged at FP1, FP2, AF5, and AF6 (Fig. 1). EEG signals were segmented into 4-s time series with 50% overlapping epochs. All filters used a zero-phase finite impulse response to prevent changes in phase. In addition, the correction algorithm employing nonlinear signal decomposition was used to correct the amplitude and base line of the signals. Calculations were performed after removal of signals outside the range of biological noise and EEG signals. Because the amplitude of noise is also physiologically significant, we used correction methods to limit the elimination of noise. However, the calculation was not performed if the data exceeded 50% of the epoch. PLEM™ operates by extracting and combining EEG signals from the frontal and prefrontal regions. The instantaneous phase was extracted via Hilbert transform using the signal processing toolbox in MATLAB (version 2017b, Mathworks Inc., Co., Ltd., USA). The PLE value was quantified using the entropy of regularity or irregularity in the temporal variation of phase difference between two EEG signals. In order to calculate the PLE value, the instantaneous phase signal was extracted from two signals and the difference value of the instantaneous phase was encoded. *S*_*t*_ = 1 if *∆ϕ*_*t*_ > 0 (i.e., first signal is phase leading the second signal), and *S*_*t*_ = 0 if *∆ϕ*_*t*_ < 0 (i.e., first signal is phase lagging the second signal). Thus, the vector *S*_*t*_, representing the temporal pattern of the phase relationship is given by

1 $$ {S}_t=\left\{\ {s}_t,{s}_{t+\tau },\dots {s}_{t+\left(m-1\right)\tau }\ \right\}t=1,2,\dots, N-\left(m-1\right)\tau $$

where, *m* and *s* represent pattern size (word length) and time lag, respectively. For example, with *m* = 3, eight patterns (“000,” “001,” “010,” “100,” “011,” “101,” “110,” and “111”) can be generated. Finally, the PLE value was calculated by applying the standard Shannon entropy formula for the distribution of the phase patterns:

2 $$ \mathrm{PLE}=-\frac{\sum {p}_j\mathit{\log}{p}_j}{\mathit{\log}\left({2}^m\right)} $$

In Eq. (2), p_*j*_ represents the probability of the occurrence of the *j*^th^ pattern in a given input signal, and m represents the size of one pattern. Eq. (2) is in the form of a fraction, where the numerator is the entropy of the probability of different phase patterns occurring in the signal, and the denominator is the number of all possible patterns. The normalization term in the denominator scale of the PLE value is the range [0 1]. PLEM™ displays the index value on the screen in a linear scale (× 100) with a value between 0 and 100. The PLE is an algorithm designed to reflect the functional connectivity of the frontal area in the brain. In the awake state, the histogram distribution of patterns is relatively even and thus has a high PLE value. In the sedated state, the distribution of patterns is biased toward a low PLE value.

### Spectral analysis

We used spectral analysis to analyze whether the EEG signal acquired from PLEM™ was consistent with the typical known patterns in the persistence and power spectrograms during propofol anesthesia [7, 12].

The persistence spectrogram was analyzed using MATLAB. We divided the EEG signal into segments with a uniform epoch length (4 s), then overlapped the spectrogram (2 s) such that the frequency power at each frequency (0.125 Hz) represented a high percentage of the spectrogram. EEG signals were divided into three states during propofol anesthesia: awake state (A-state), unconscious state (UC-state), and recovery of consciousness state (ROC-state). The frequency and frequency power were plotted on the x-axis and y-axis, respectively. The distribution of power was shown using color to visually represent a decibel ratio (%) [21].

For the power spectrogram, we estimated the standard Multitaper Power Spectral Density (MPSD) using MATLAB [22]. For computing the power spectrogram, we obtained individual four-channel EEG signals acquired from PLEM™. We computed four-channel median spectrograms by taking the median across all time epochs. The time was plotted on the x-axis and frequency on the y-axis; the signal frequency power was expressed in scale color. The power spectrogram quantifies the frequency distribution of energy or power within the EEG signal over time. We calculated the MPSD using 8-s EEG segments (4 s before to 4 s after each EEG measurement point) to quantify the frequency power ratio for a given propofol Ce. We set the following parameters: window length (2 s), overlap (1 s), time-half bandwidth product (3 Hz), and spectral resolution (0.25 Hz). We calculated the average of the listed four-channel MPSD values for all band power values.

### Calculation of EEG band power

We also computed the ratio of EEG band powers (gamma, beta, alpha, theta, and delta) using the following equation for each time point with propofol Ce in all patients:

$$ Ratio\kern0.17em of\; EEG\; band\kern0.17em power\;\left(\gamma, \beta, \alpha, \theta, \delta \right)=\frac{Frequency\kern0.17em band\kern0.17em power\left(\gamma, \beta, \alpha, \theta, \delta \right)}{Total\kern0.17em frequency\kern0.17em power} $$

The absolute values of EEG-derived band power in each patient were calculated using the ratio to reduce the effect of differences between patients.

### Statistical analysis

Statistical analysis was performed using SPSS software version 20 (IBM Corporation, Armonk, NY, USA). A correlation coefficient of 0.7, for the index of the anesthetic depth monitoring device according to the propofol Ce, was considered to be clinically significant. We calculated a minimum requirement of 29 patients. We also calculated a sample size of 32 based on previous observational studies correlating EEG-based anesthetic depth monitoring (a difference of 10 between BIS and entropy based on pilot study, a power of 90% with an α value of 0.05) [23]. Considering possible data loss, we decided to study 35 patients.

The data are presented as mean ± SD or median for continuous variables, and number of patients for categorical variables. Spearman correlation analysis was performed between the PLE value and propofol Ce and displayed using box and whiskers plots. Repeated measures analysis of variance (ANOVA) was performed for changes in PLE value before and after muscle relaxant injection, and for the change in EEG-band power during propofol anesthesia, displayed using box and whiskers plots. Post-hoc multiple comparisons were then performed using the Tukey test. A comparison between the PLE value and BIS value at the same points in time was performed using the *t*-test with the Bland-Altman graph. *P-*values < 0.05 were considered statistically significant.

## Results

Of the 35 patients, one was excluded because of a technical error during recording with the sensor electrode. Therefore, 34 patients were included in the statistical analysis. The demographic data of these patients are shown in Table 1.

### Correlation between PLE value and changes in propofol Ce

**Fig. 2:**
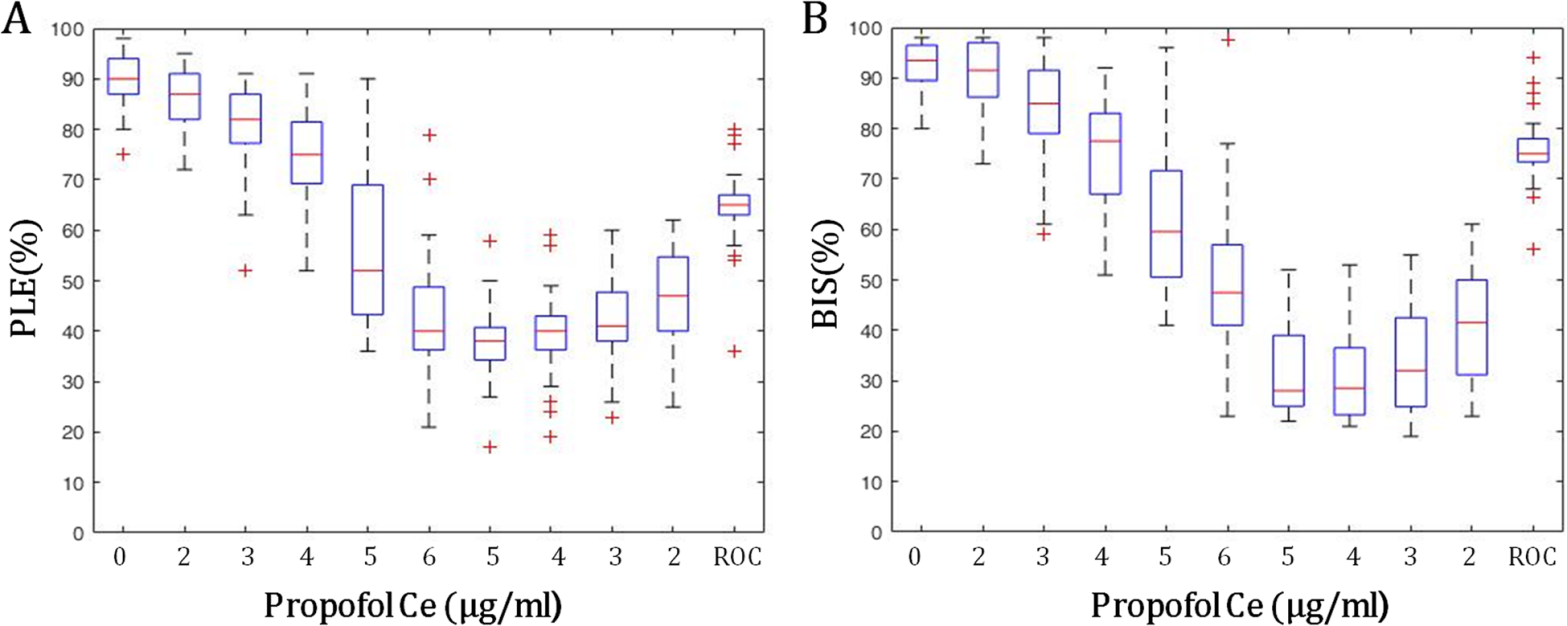
The change in PLE and BIS values during propofol anesthesia. The box and whiskers plots show PLE and BIS values at the time when a given propofol target effect concentration (propofol Ce) was reached. **a** For PLE, Spearman correlation coefficient = 0.835 (from propofol Ce 0 to propofol Ce 6.0 μg/ml). **b** For BIS, Spearman correlation coefficient = 0.781 (from propofol Ce 0 to propofol Ce 6.0 μg/ml). Abbreviations: PLE, phase lag entropy; BIS, bispectral index; LOC, loss of consciousness; ROC, recovery of consciousness. The boxes depict the median values and the 25th and the 75th percentiles (lower whisker = − 1.5 × IQR, upper whisker = + 1.5 × IQR, IQR; inter-quartile range, +; outlier)

The PLE values were inversely correlated to changes in propofol Ce (propofol Ce from 0 to 6.0 μg/ml, Spearman correlation coefficient r^2^ = − 0.835; propofol Ce from 6.0 to 2.0 μg/ml, r^2^ = − 0.467) (Fig. 2a). The PLE value at LOC was 62.3 ± 10.9 with propofol Ce 4.4 ± 0.8 μg/ml, whereas the PLE value at ROC was 64.4 ± 9.6 with propofol Ce 1.0 ± 0.2 μg/ml.

### Spectral analysis of EEG acquired from PLEM™

**Fig. 3:**
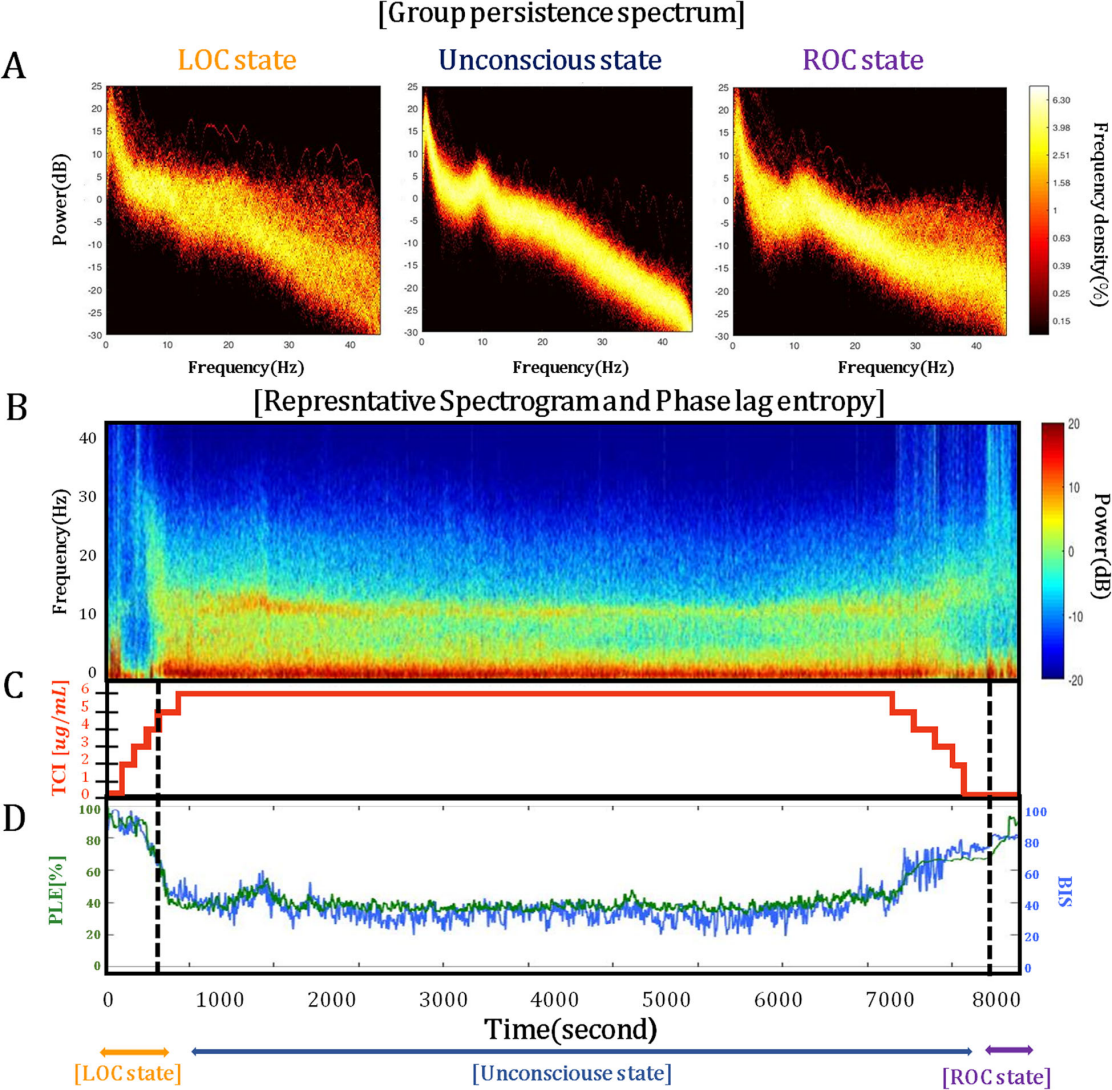
The spectrograms obtained from PLEM™ during propofol anesthesia. **a** Group-level persistence spectrogram for different states of consciousness (LOC-state, UC-state, and ROC-state). **b** Representative power spectrogram showing the power of the slow and delta (0.1 to 4 Hz) and alpha (8 to 13 Hz) band oscillations, and **c** the time domain phase lag entropy (PLE) obtained from PLEM™ during propofol anesthesia. Abbreviations: TCI, target-controlled infusion; LOC, loss of consciousness; UC, unconsciousness; ROC, recovery of consciousness. The black dashed line indicates when LOC and ROC occurred

**Fig. 4:**
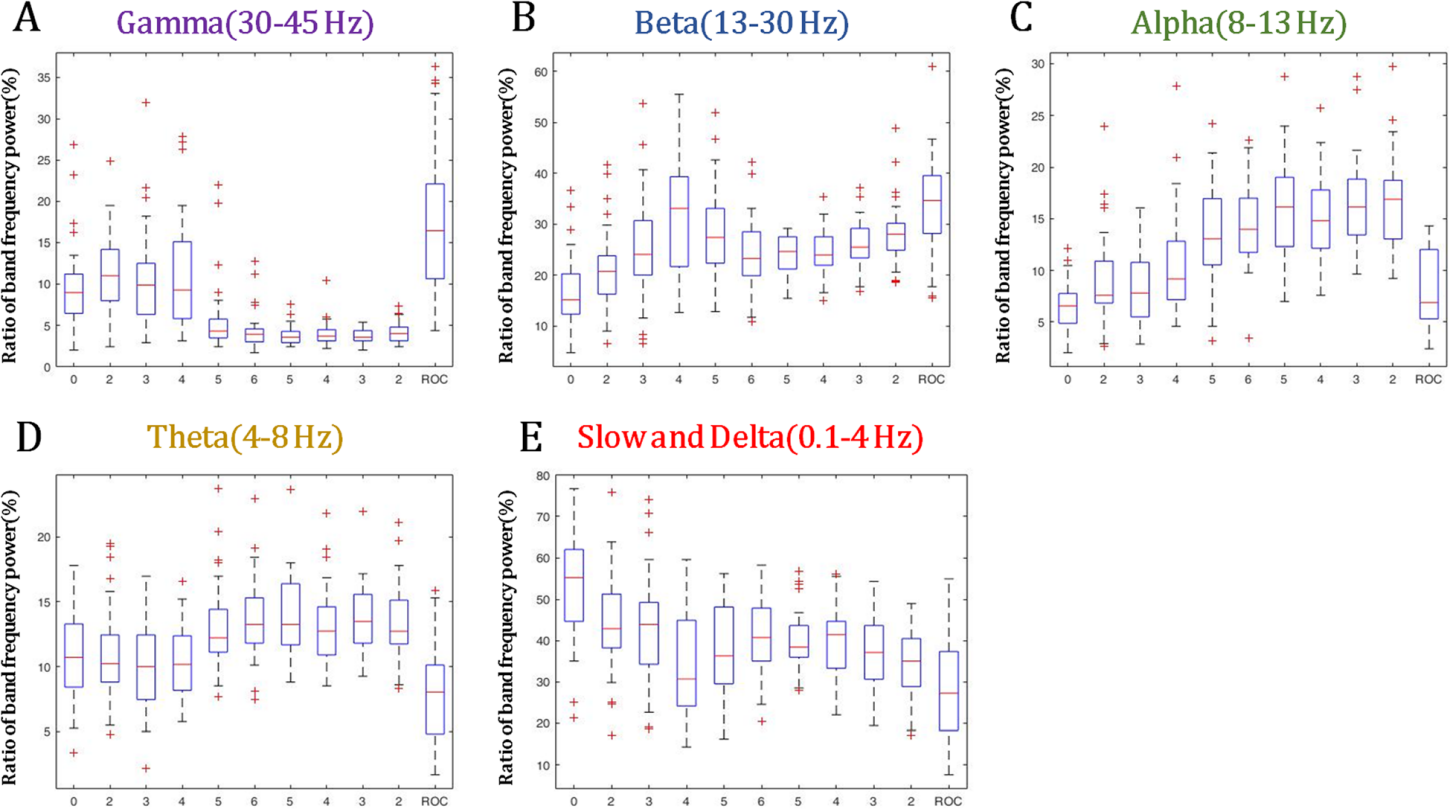
The change in the ratio of electroencephalogram (EEG) band power during propofol anesthesia. The box and whiskers plots show the ratio of EEG band power (gamma, beta, alpha, theta, and delta) at the time when a given propofol target effect concentration (propofol Ce) was reached. The boxes depict the median values and the 25th and the 75th percentiles (lower whisker = − 1.5 × IQR, upper whisker = + 1.5 × IQR, IQR; inter-quartile range, +; outlier)

The persistence spectrogram showed a wide distribution of power for a given frequency in the LOC-state and ROC-state compared to the dense distribution of power for a given frequency in the UC-state (Fig. 3a). The power spectrogram showed a typical pattern of propofol-induced sedation with slow alpha (8–12 Hz) band oscillations (Fig. 3b) [7]. PLE and BIS values were inversely proportional to propofol Ce (Fig. 3c). For each EEG band power, as propofol Ce increased, the alpha-, beta-, and theta-band powers showed a tendency to increase, and the gamma-band power showed a tendency to decrease. There was no significant change in the delta-band power. However, the changes in EEG band power were not statistically significant because of the wide range of SD and some outliers (Fig. 4).

### A comparison of PLE and BIS

**Fig. 5:**
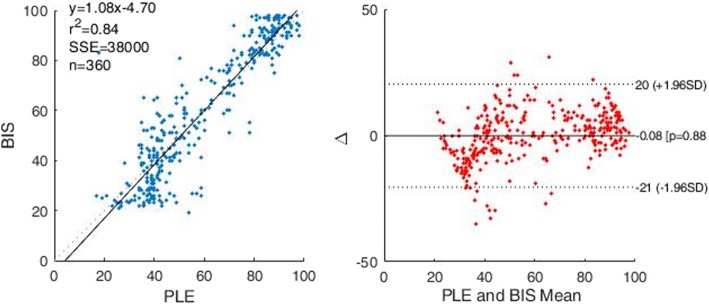
A comparison of PLE and BIS using the Spearman correlation graph and Bland-Altman graph. Abbreviations: PLE, phase lag entropy; BIS, bispectral index; SD, standard deviation

The PLE value demonstrated a strong correlation with the BIS value during the change in propofol Ce from 0 to 6.0 μg/ml (r^2^ = 0.84) (Fig. 5). The PLE was significantly higher than the BIS at all propofol Ce values prior to intubation (*P* < 0.05), and lower than the BIS at all propofol Ce values after intubation (P < 0.05) (Table 2) (Fig. 5). The PLE values were similar to the BIS at LOC (PLE: 62.3 ± 10.9, BIS: 61.8 ± 10.5), but lower at ROC (PLE: 64.4 ± 9.6, BIS: 75.7 ± 6.4) (P < 0.05).

**Table 2:** PLE and BIS values at the time point when propofol reached the target effect concentration

Propofol Ce (μg/ml)	PLE	BIS	*P*-value
0	89.1 ± 8.5*	91.6 ± 5.7	0.042
2	83.9 ± 12.7*	90.7 ± 6.4	0.002
3	79.0 ± 10.7*	83.5 ± 10.7	0.000
4	74.5 ± 9.2	74.9 ± 11.1	0.798
5	56.8 ± 14.8*	62.0 ± 14.8	0.001
6	43.7 ± 11.4*	49.7 ± 14.5	0.002
Intubation			
5	37.3 ± 7.1*	31.6 ± 8.2	0.005
4	39.3 ± 7.8*	30.8 ± 8.8	0.000
3	41.7 ± 9.1*	34.5 ± 10.7	0.000
2	47.2 ± 9.1*	41.1 ± 11.6	0.000

Data are mean ± standard deviation or values. Abbreviations: PLE, phase lag entropy; BIS, bispectral index; propofol Ce, propofol target effect-site concentration. * *P*-value < 0.05 compared with BIS at the same time points for propofol Ce 0–6 μg/ml

### Effect of muscle relaxants

Both PLE and BIS values significantly decreased after injection of the muscle relaxant (PLE: 43.8 ± 11.6 vs 38.3 ± 4.2, BIS: 49.7 ± 14.5 vs 36.5 ± 9.7) (P < 0.05).

## Discussion

In this study, PLE values from PLEM™ were inversely correlated to changes in propofol Ce during the induction and emergence period of propofol anesthesia. The persistence spectrogram and power spectrogram using EEG signals acquired from the PLEM™ were consistent with the typical known patterns seen in propofol anesthesia. The PLEM™ was similarly comparable for anesthetic depth monitoring with BIS™ during the change of propofol Ce 0 to Ce 6.0 μg/ml. The PLE value was lower at BIS values above 40, but higher at BIS values below 40. The PLE and BIS values were similar at LOC, but the PLE values were smaller than the BIS values at ROC.

To date, there have been many studies for developing a method of assessing anesthetic depth using processed EEG data [8]. The most widely used processed EEG-based anesthetic depth monitor, BIS™, quantifies the consistency of phase-coupling and frequency of single-channel EEG in the brain, whereas PLEM™ quantifies the entropy based on the spatial or connectivity information of four-channel EEG signals by measuring the regularity of variations in the temporal phase difference between two separate areas of the brain [4, 8].

PLEM™ is a recently developed anesthetic depth monitoring device that uses four-channel EEG [4]. The PLE value in PLEM™ is composed of three sub-parameters, PLE1 (8–32 Hz), PLE2 (0.1–1 and 32–45 Hz), and BSR (2–32 Hz). PLE1 (light hypnotic state) is calculated from the alpha (8–13 Hz) and beta (13–30 Hz) bands, whereas PLE2 (deep hypnotic state) is calculated from the slow-frequency (0.1–1 Hz) and gamma (30–45 Hz) bands. BSR is composed of two types of burst-suppression detection, such as the portions of the isoelectric EEG and/or a very low power frequency. The PLE value (scale 0–100) is calculated by combining PLE1, PLE2, and BSR with appropriate weights.

In this study, we found that the PLE values were inversely correlated to the changes in propofol Ce when propofol Ce was increased (r^2^ = − 0.835) and decreased (r^2^ = − 0.467). The PLE values at LOC and ROC were similar (62.3 and 64.4, respectively). Koo et al.’s study [24] results of propofol Ce at LOC and ROC (4.4 ± 1.1 μg/ml, 1.1 ± 0.3 μg/ml) are similar to that in our study. In Lee et al.’s study [4] that compared recently used anesthetic depth monitors, the PLE value exhibited the highest agreement with the level of consciousness (using the modified OAA/S score) relative to other monitors such as the BIS, relative beta ratio (RBR), approximate entropy (ApEn), and permutation entropy (PeEn) values. Recently, clinical studies have been reported for PLEM™ during propofol-induced sedation [18–20]. Jung et al. [20] reported that PLEM™ was comparable with BIS™ in correlational studies using the OAA/S score during propofol-induced sedation (Spearman’s Rho: 0.755 for PLE, 0.788 for BIS). Ki et al. [18] also reported the pharmacodynamic modeling for each OAA/S score using PLE values from the PLEM™ (Ce_50_ value: 1.67 μg/ml, 1.96 μg/ml, 2.22 μg/ml, and 2.69 μg/ml for OAA/S scores of ≤4, ≤3, ≤2, and ≤ 1, respectively). Therefore, based on the above study results, the PLEM™ may be used for monitoring anesthetic depth during propofol anesthesia.

Propofol reduces the excitation potential input to the cortex by binding to post-synaptic γ-aminobutyric acid A (GABA_A_) receptors [8, 25]. Modeling and experimental studies using propofol suggest that the potentiation of GABA receptors leads to a state of thalamo-cortical synchrony associated with unconsciousness, observed as frontal slowing and alpha band oscillations [8]. In the states bordering consciousness, such as LOC, the power of alpha and beta bands in EEG waves move from the occipital region to the frontal region. This is a recognized phenomenon called “anteriorization” [7, 8, 15, 26]. Coherent alpha oscillations and disruption of neural spiking activity associated with slow oscillations are the two main mechanisms of propofol-induced sedation [7, 27, 28].

We used spectral analysis of the persistence spectrogram and power spectrogram to see whether the EEG signal acquired from PLEM™ reflected typical known patterns of propofol anesthesia that had been reported in previous studies [7, 12, 29]. The persistence spectrogram showed wide distribution in the LOC-state and ROC-state, in contrast to a dense distribution in the UC-state. The power spectrogram also showed a typical pattern with slow alpha (8–12 Hz) band oscillations [7].

Using PLEM™, we could measure EEG band power (the ratio of gamma, beta, alpha, theta, and delta waves). In previous studies of EEG band power during propofol-induced sedation, it was found that as the modified OAA/S score decreases, the power of the alpha-beta band gradually increases, and the delta-band power increases after LOC [7, 12, 30]. We also observed the same results in our study; as propofol Ce increased, alpha-, beta-, and theta- band powers showed the tendency to increase, and gamma-band power showed the tendency to decrease. However, these changes were not statistically significant because of the wide SD and some outliers. Generally, as the anesthetic depth increases, the frequency of the EEG band power shifts toward the lower frequencies (beta to alpha to theta to delta). If the propofol Ce was increased any further in our study, burst suppression activity would ensue [7]. In our study, the gamma-band power increased during the start of propofol TCI and then decreased at propofol Ce 4–5 μg /ml; this might be because of noise interference with the EEG [25].

Before the start of our study, by prioritizing spatial concepts using four-channel EEG in PLEM™, we hypothesized that the PLE value might reflect the anesthetic depth better than the other widely used anesthetic depth monitors, such as the BIS value. In our study, the PLE value demonstrated a strong correlation with the BIS value. The PLE was significantly higher than the BIS prior to intubation, and lower than the BIS after intubation. The PLE values were similar to the BIS at LOC (PLE: 62.3, BIS: 61.8), but lower to the BIS at ROC (PLE: 64.4, BIS: 75.7). In the previous studies [31–33], the BIS values were affected by the degree of neuromuscular block. Contrary to the BIS, the PLE is less influenced by EMG signal. In PLE algorithm, noise introduced into both channel (R1, R2) at the same time is removed during the binarization process (“000,” “001,” “010,” “100,” “011,” “101,” “110,” and “111”) of phase difference by phase extraction. The EMG activity in our result was higher in ROC compared to LOC (27.9 ± 41.7% vs. 67.4 ± 22.5%). We suspect the observed differences between the BIS and PLE, especially during ROC phase, may be due to the difference in the EMG-EEG interferences of both devices. However, in the results, both PLE and BIS values significantly decreased after injection of the muscle relaxant. Further studies are suggested for the effect of EMG signals on the PLE and the BIS.

The limitations of our study are as follows. First, BIS sensor (circle 3) was slightly mal-positioned (the commercially recommended location of the BIS sensor is as follows: circle 1 at the center of the forehead, approximately 4 cm above the nose, circle 2 at 2.8 cm lateral right to circle 1, and circle 3 on the temple area between the corner of the eye and the hairline). Second, the disagreement between the PLE value and the BIS value at each time point showed large standard deviation. Third, the PLE and BIS values were not measured at the same point in time because of the different smoothing rates of the devices. The smoothing rates for PLE and BIS were 4 s and 10 s, respectively. Fourth, PLE and BIS values changed continuously after reaching propofol Ce. The inter-trial variability of these values was related to time. Fifth, we only investigated the performance of PLEM™ in young adults during propofol anesthesia [7]. Further studies are needed to validate the PLE value from PLEM™ for pediatric or geriatric patients and with other drugs such as other GABAnergic anesthetic drugs and non-GABAnergic drugs such as ketamine, dexmedetomidine, and N_2_O.

## Conclusion

The PLE value obtained using PLEM™ is a useful anesthetic depth indicator, similar to the BIS value, in patients subjected to propofol anesthesia. Spectral analysis of the raw EEG signals acquired from the PLEM™ demonstrated the typical patterns of propofol anesthesia.

## Data Availability

The dataset generated and analyzed during the current study is available from the corresponding author on reasonable request.

## Abbreviations

ANOVA: Analysis of variance
ApEn: Approximate entropy
ASA: American Society of Anesthesiologists
A-state: Awake state
BIS: Bispectral index
Ce: Target effect-site concentration
EEG: Electroencephalography
EMG: Electromyography
GABA: γ-aminobutyric acid
LOC: Loss of consciousness
MPSD: Multitaper power spectral density
OAA/S: Observer’s Assessment Alertness/Sedation
PeEn: Permutation entropy
PLE: Phase lag entropy
RBR: Relative beta ratio
ROC: Recovery of consciousness
ROC-state: Recovery of consciousness state
SQI: Signal quality index
TCI: Target-controlled infusion
UC-state: Unconscious state
